# Validation of Androgen Receptor loss as a risk factor for the development of brain metastases from ovarian cancers

**DOI:** 10.1186/s13048-020-00655-2

**Published:** 2020-05-04

**Authors:** Gloria Mittica, Margherita Goia, Angela Gambino, Giulia Scotto, Mattia Fonte, Rebecca Senetta, Massimo Aglietta, Fulvio Borella, Anna Sapino, Dionyssios Katsaros, Furio Maggiorotto, Eleonora Ghisoni, Gaia Giannone, Valentina Tuninetti, Sofia Genta, Chiara Eusebi, Marina Momi, Paola Cassoni, Giorgio Valabrega

**Affiliations:** 1 Unit of Oncology, ASL Verbano Cusio Ossola (VCO), Domodossola, Italy; 2 Unit of Pathology, Città della Salute e della Scienza, Turin, Italy; 3 Department of Medical Sciences, University of Turin, Turin, Italy; 4 Department Obstetrics and Gynecology, University of Brescia, Brescia, Italy; 5 Department of Oncology, University of Torino, Turin, Italy; 6 Candiolo Cancer Institute, FPO - IRCCS, Candiolo, TO Italy; 7 Department of Surgical Sciences, Gynecology, AOU, Città della Salute e della Scienza, Turin, Italy

**Keywords:** Ovarian cancer, Brain metastases, Androgen receptor, Immunohistochemistry

## Abstract

**Background:**

Central nervous system (CNS) spreading from epithelial ovarian carcinoma (EOC) is an uncommon but increasing phenomenon. We previously reported in a small series of 11 patients a correlation between Androgen Receptor (AR) loss and localization to CNS. Aims of this study were: to confirm a predictive role of AR loss in an independent validation cohort; to evaluate if AR status impacts on EOC survival.

**Results:**

We collected an additional 29 cases and 19 controls as validation cohort. In this independent cohort at univariate analysis, cases exhibited lower expression of AR, considered both as continuous (*p* <  0.001) and as discrete variable (10% cut-off: *p* <  0.003; Immunoreactive score: *p* <  0.001). AR negative EOC showed an odds ratio (OR) = 8.33 for CNS dissemination compared with AR positive EOC. Kaplan-Meier curves of the combined dataset, combining data of new validation cohort with the previously published cohort, showed that AR <  10% significantly correlates with worse outcomes (*p* = 0.005 for Progression Free Survival (PFS) and *p* = 0.002 for brain PFS (bPFS) respectively).

Comparison of AR expression between primary tissue and paired brain metastases in the combined dataset did not show any statistically significant difference.

**Conclusions:**

We confirmed AR loss as predictive role for CNS involvement from EOC in an independent cohort of cases and controls. Early assessment of AR status could improve clinical management and patients’ prognosis.

## Background

Epithelial ovarian cancer (EOC) is the third cause of death among gynaecological malignancies worldwide [1].

Peritoneal cavity is the most common EOC site of progression, but haematogenous spreading may occur too [2], involving liver, distant lymph nodes, pleura, lungs and, less frequently, skin and bone [3, 4]. Central nervous system (CNS) is a rare localization of disease, with a reported incidence ranging between 0,3 and 12% [4, 5]. Recently, brain involvement seems to increase [6], probably due to improvement of medical treatments, radiotherapy and surgery [3, 7–10]. Negative prognostic factors in patients with CNS involvement are extra-cranial disease, single-treatment approach, multiple brain lesions, low Karnofsky Performance Status, non-serous histology, older age at diagnosis [7–10] and non-surgical treatment [11]. Median overall survival (OS) is about 5 months for women undergoing a single-modality treatment versus 22 months if a multimodal approach is pursued [10, 11].

Unfortunately, no predictive biomarkers of CNS involvement have been validated in EOC at present.

Considering its already known negative prognostic value, we focused our interest on Androgen Receptor (AR) [12, 13]. Interestingly, in our previously published small series of 11 patients, AR-negativity predicted a 9.5 times higher propensity of CNS involvement [14].

Aims of the present study were: i) to confirm AR’s predictive role for CNS dissemination from EOC in an independent validation cohort, and ii) to assess the role of AR expression in terms of progression free survival (PFS) and brain progression free survival (bPFS) in the combined dataset.

## Methods

### Patients’ validation cohort collection

In order to validate AR’s predictive role of CNS involvement form EOC, we extended our previously published series of eleven patients [14]. Fourteen new cases with paired brain lesions and 15 new cases with CNS involvement not surgically removed, diagnosed from April 2000 to December 2015, were added. These cases were obtained from the Pathology archives of Hospitals with recognized expertise in treatment of EOC, members of the Neuro-Oncological Network of the Piedmont Region (Italy) (AOU Città della Salute e della Scienza of Turin, AO San Giovanni Bosco Hospital of Turin, AOU Maggiore della Carità of Novara, AO S. Croce e Carle Hospital of Cuneo, AO SS. Antonio, Biagio e Cesare Arrigo of Alessandria, ASL CN2 Hospitals of Alba and Bra, AO Martini, Hospital of Turin, AO Maria Vittoria, Hospital of Turin, AO Mauriziano, Hospital of Turin),and from Azienda Socio Sanitaria Territoriale (ASST) Spedali Civili of Brescia, Lombardy Region (Italy).

Inclusion criteria were: i) clinical evidence of CNS progression by radiological imaging; ii) access to follow-up data; iii) availability of histological blocks of at least the primary ovarian tumor.

For each case the following clinical and pathological parameters were collected: i) age at diagnosis; ii) date of diagnosis of both primary ovarian cancer and CNS metastasis; iii) morphological and histological features of both ovarian tumor and brain metastasis (histotype and grade); iv) FIGO stage at diagnosis and residual tumor after surgery; v) date and site of first relapse; vi) treatments received at diagnosis and relapse; vii) date of death or last follow-up; viii) overall survival (OS), counted as the time from the date of EOC diagnosis to the date of death or last follow-up; ix) progression free survival (PFS) estimated as the time from EOC diagnosis to the date of first clinical relapse; x) progression brain metastasis free survival (bPFS) calculated as the time from primary tumor diagnosis to brain metastasis development; xi) brain metastasis overall survival (bOS) determined as the time from the date of brain metastasis diagnosis to death or last follow-up. Nineteen new controls, diagnosed from June 2011 to June 2016, without CNS progression but with similar clinical and histopathological features and follow up were selected from the clinical records of Candiolo Cancer Institute (FPO-IRCCS).

The study was submitted to and approved by the Ethic Institutional Review Board for “Biobanking and use of human tissues for experimental studies” of the Pathology Service of the AOU Città della Salute e della Scienza (Turin, Italy). The project provided a verbal and not written informed consent from the patients due to the retrospective approach of the study, which did not impact on their treatment. All the cases were anonymously recorded. The Institutional Review Board approved this consent procedure.

### Immunohistochemistry procedures

Haematoxylin and eosin (H&E) slides were assessed and the most representative paraffin block of each lesion was selected for immunohistochemistry (IHC). A three-micrometer-thick section was collected on a SuperFrost Plus slide and, IHC reaction against AR (monoclonal antibody, clone SP107, pre-diluted in Tris Buffer, pH 7.3–7.7, with 1% BSA and <  0.1% Sodium Azide, Ventana, Roche) was performed on an automated immunostainer (VentanaBenchMark XT AutoStainer, Ventana Medical Systems, Tucson, AZ, USA). Nuclear staining was considered positive. Prostate tissue was used as positive control, while a triple negative basal-like breast cancer sample was used as negative control.

Two observers, blinded to clinical data, independently evaluated staining results, counting the percentage of positive neoplastic nuclei on 10 high-magnification fields (400x). At least 100 fields were counted for each sample. The intensity of nuclear staining was also estimated with a score ranging from 0 to 3+ (0: absent staining; 1+: weak nuclear staining; 2+: moderate nuclear staining; 3+: intense nuclear staining). Androgen receptor was considered as follows: i) continuous variable (ratio of positive neoplastic cells from 0 to 100%), ii) discrete variable using cut-off values of 1% [15] and 10% [13, 16], as previously reported in literature, and iii) dichotomized variable according to Immunoreactive Score (IRS) [12]. The IRS was obtained by multiplying the intensity of staining (from 0 to 3+) by the percentage of positive cells (0 = 0% of stained nuclei; 1 = < 10% of stained nuclei; 2 = 10–50% of stained nuclei; 3 = 51–80% of stained nuclei; 4 = > 80% of stained nuclei). The samples were considered positive or negative as follows: score ≤ 2: negative; score > 2: positive.

### Statistical analysis

All statistical analyses were performed using SPSS software for Windows (version 22.0; SPSS Inc., Chicago, IL). Categorical variables were initially compared with Pearson Chi-square test, but when results were not reliable, Fisher exact test has been considered for further statistical analyses. Continuous variables were compared using analysis of variance (ANOVA) or dependent T test for paired samples. Kaplan-Meier curves were drawn to analyse survival outcome using the log-rank test method. *P* values < 0.05 were considered significant, and all tests were two-tailed.

## Results

### Validation cohort

We collected 29 new cases and 19 new controls as validation cohort.

#### Case dataset

Median age at diagnosis was 57 years (range 39–76); the majority of cases were categorized as serous carcinomas (25/29, 86%) and all cases were high grade (G3) (29/29, 100%). According to FIGO classification, 2 cases (7%) were stage II, 19 (65.5%) stage III and 8 (27.5%) stage IV.

Twenty-two out of 29 cases (76%) were treated with up-front surgery; seven (24%) received neo-adjuvant chemotherapy (NACT) followed by Interval Debulking Surgery (IDS). Residual tumor was present in 18 cases (62%), absent in 11 (38%).

All cases relapsed, with a median PFS of 15 months (range 0–62). Median age at diagnosis of CNS involvement was 59 years, with a median bPFS of 25 months (range 0–87 months). Fourteen out of 29 (48%) patients developed brain metastases as the first site of relapse.

Overall, median bOS was 17 months (range 2–112), while median OS was 48 months (range 4–173). At the time statistical analyses were performed (May 2018), 7/29 patients (24%) were alive, while 22 (76%) had died.

Table 1 shows the most relevant clinico-pathological parameters of cases subgroup of the validation cohort.

**Table 1:** Clinico-pathological features of primary ovarian lesion in validation cohort: case dataset vs control dataset

**Clinico-histopathological features validation cohort**	**Case Dataset** ***n*** **= 29 (%)**	**Control Dataset** ***n*** **= 19 (%)**	***p*Value**
**Age, median (years) [range]**	57 [39–76]	52 [40–78]	N.S.
**Histological type**			
Serous	25 (86)	19 (100)	N.S.
Clear cell	2 (7)		
Undifferentiated	1 (3)		
Other	1 (3)		
**Histological grade**			
G3	29 (100)	19 (100)	N.S.
**FIGO stage**			
II	2 (7)	0 (0)	N.S.
III	19 (65.5)	13 (68)	
IV	8 (27.5)	6 (32)	
**Type of surgery**			
Upfront	22 (76)	8 (42)	0.02
Neoadjuvant CT + IDS	7 (24)	11 (58)	
**Macroscopic residual tumor**			
Present	18 (62)	6 (32)	0.04
Absent	11 (38)	13 (68)	
**First-line chemotherapy**			
Platinum-based	29 (100)	19 (100)	N.S.
**Relapse**			
Present	29 (100)	12 (63)	0.0004
Absent		7 (37)	
**First site of relapse**			
CNS	14 (48)	0 (0)	0.003
Lymph nodes and / or peritoneum	11 (38)	11 (92)^a^	
Other	4 (14)	1 (8)^a^	
**Patient’s status**			
Alive	7 (24)	15 (79)	0.0002
Dead	22 (76)	4 (21)	
**PFS, median (months) [range]**	15 [0–62]	17 [10–62]	N.S.
**OS, median (months) [range]**	48 [4–173]	32 [19–78]	N.S.

*IDS* Interval Debulking Surgery, *CT* Chemotherapy, *CNS* Central Nervous System, *PFS* Progression Free Survival, *OS* Overall Survival, *N.S*. not significant

^a^Disease progression did not occur for 7 patients

For more detailed features of CNS involvement in the combined dataset see Table 2.

**Table 2:** Clinical parameters of the 40 CNS metastases included in the study

**Cases**	**Age**	**Neurological symptoms**	**N° of lesions**	**Site of lesions**	**Treatment of CNS metastases**	**bPFS (months)**	**bOS (months)**
**Previously Published Cohort**							
**1**	49	NA	Single	Parietal	Surgery, CT	11	6
**2**	57	Drowsiness	Single	Occipital	Surgery	68	42
**3**	70	Ataxia	Single	Parietal, occipital	Surgery, WBRT	29	41
**4**	70	Unilateral symptoms	Single	Parietal	Surgery, CT, WBRT	22	6
**5**	50	Headache, vertigo	Double	Parietal, occipital, frontal	Surgery, CT, WBRT	18	29
**6**	70	Aphasia, disorientation, dizziness	Single	Parietal	Surgery, SRS	28	56
**7**	52	NA	Single	Frontal	Surgery, CT, WBRT	54	7
**8**	62	Headache, altered walking gait	Multiple	Temporal, frontal, occipital	Surgery, WBRT	15	3
**9**	46	NA	Single	NA	Surgery	23	3
**10**	72	Ataxia, dysmetria	Multiple	Frontal (major)	Surgery, WBRT	18	7
**11**	74	Vertigo	Single	Frontal	Surgery, WBRT	25	64
**Validation Cohort**							
**12**	56	Seizures, aphasia, altered walking gait	Single	Frontal	Surgery, WBRT	0	48
**13**	77	Persistent vomiting	Single	Cerebellar	Surgery	26	40
**14**	79	Dysarthria, altered walking gait	Single	Parietal	Surgery	36	6
**15**	66	Paresthesia, dizziness	Multiple	Parietal, occipital	Surgery, CT, SRS	30	34
**16**	69	Seizures, dysmetria	Single	Frontal	Surgery	0	12
**17**	67	Headache, diplopia	Single	Occipital	Surgery, CT	45	12
**18**	67	Headache, vomit	Single	Frontal	Surgery, WBRT	22	40
**19**	68	Headache	Single	Frontal	Surgery, WBRT	26	27
**20**	56	Aphasia, seizures, dysarthria, dysmetria	Multiple	Temporal, parietal, occipital	Surgery, CT, WBRT	61	112
**21**	57	Headache, vomit	Single	Cerebellar	Surgery, WBRT	18	41
**22**	60	Altered walking gait	Multiple	Cerebellar, supratentorial	Surgery, CT, WBRT	19	13
**23**	54	Headache, paresthesia, hemiparesis	Multiple	Temporal, parietal	Surgery, CT, WBRT	15	44
**24**	46	Altered walking gait	Single	Cerebellar	Surgery, WBRT	25	8
**25**	69	Altered walking gait, dizziness, Hemianopia	Multiple	Parietal, occipital	Surgery, WBRT	22	39
**26**	71	Headache	Double	Frontal, Cerebellar	WBRT, CT	32	27
**27**	51	Aphasia, verbal amnesia	Multiple	Frontal, temporal, occipital	WBRT	34	6
**28**	59	Headache, altered walking gait	Multiple	Cerebellar, supratentorial	WBRT	25	18
**29**	74	Altered walking gait, diplopia	Multiple	Cerebellar, parietal, temporal	WBRT	87	17
**30**	58	Seizures	Multiple	Cerebellar, supratentorial	WBRT	16	22
**31**	78	NA	Multiple	supratentorial	WBRT	27	2
**32**	60	NA	Double	Temporal, frontal	WBRT, CT	36	44
**33**	75	Asthenia, fatigue	Multiple	Cerebellar, supratentorial	WBRT, CT	21	3
**34**	51	Headache	Multiple	Parietal, occipital	WBRT, CT	72	8
**35**	54	NA	Single	Occipital	WBRT	1	3
**36**	49	Hemiparesis	Multiple	Frontal, temporal, occipital	SRS	28	51
**37**	39	Paresthesia, Dysarthria	Multiple	Occipital, cerebellar	WBRT, CT	4	4
**38**	55	Drowsiness	Multiple	Parietal, frontal	/	38	2
**39**	57	Headache, Paresthesia	Multiple	Frontal, parietal, occipital	WBRT, CT	1	15
**40**	55	Headache	Multiple	Frontal, cerebellar	WBRT, CT	11	9

*NA* not available, *WBRT* Whole Brain RadioTherapy, *SRS* Stereotactic RadioSurgery, *CT* Chemotherapy, *bPFS* Progression Brain Metastasis Free Survival, *bOS* brain metastases Overall Survival

#### Control dataset

The median age of primary EOC diagnosis was 52 years (range 40–78). All our controls were high grade (G3) serous carcinomas (19/19, 100%). There were 13 FIGO stage III (68%) and 6 FIGO stage IV (32%). Eleven out of 19 patients (58%) were treated with neoadjuvant chemotherapy followed by IDS. Up-front surgery was chosen for 8 women (42%), followed by adjuvant chemotherapy. All patients received platinum-based chemotherapy (19/19, 100). Thirteen patients (68%) had no residual tumor after surgery. Twelve controls (63%) experienced at least one relapse of the disease. Median PFS was 17 months (range 10–62), whereas median OS was 32 months (range 19–78). When data were processed, 4 (21%) patients had died.

Table 1 shows the most relevant clinico-pathological parameters of control subgroup.

Cases and controls of the validation cohort were comparable: no statistical difference was observed for age, histotype, tumor grade, FIGO stage, and first line chemotherapy (see Table 1). Significant differences between two group were observed for upfront surgery vs interval cytoreductive surgery, absence/presence of residual tumor after surgery, incidence of relapse and number of surviving patients.

### AR expression in cases and controls of the validation cohort

Immune-histochemical stainings were performed on both cases and controls of the validation cohort. Table 3 shows comparisons between cases vs controls, considered as continuous variables.

**Table 3:** Immune-histochemical results and statistical analyses of AR considered as continuous variable: comparisons of cases vs controls in validation cohort

**IHC parameter**	**Cases vs controls** **(%)**	**N**	**Mean (%)**	**Median (%)**	**Range**	***p*Value**
**AR**	Cases	29	13.21	5	0–70	**< 0.001**
	Controls	19	43.21	40	1–95	

*AR* Androgen Receptor

For AR protein expression in cases and controls of the validation cohort as dichotomized variable see Table 4.

**Table 4:** Immune-histochemical results and statistical analyses of AR considered as dichotomized variable as for both cases and controls validation datasets

**IHC parameter**	**Cut-off**	**Case dataset (primary ovarian lesions) (%)**	**Control dataset (primary ovarian lesions) (%)**	**p Value (Cases vs controls: IHC comparison)**	**OR (Cases vs controls) (CI 95%)**
**AR**	< 1%	4/29 (13.8)	0/19 (0)	0.142	
	≥ 1%	25/29 (86.2)	19/19 (100)		
	< 10%	20/29 (69)	4/19 (21.1)	**0.003**	**8,33 (2.15–32.29)**
	≥ 10%	9/29 (31)	15/19 (78.9)		
	IRS ≤ 2	24/29 (82.8)	4/19 (21.1)	**< 0.001**	
	IRS > 2	5/29 (17.2)	15/19 (78.9)		

*AR* Androgen Receptor

### AR expression is significantly reduced in cases vs controls’ validation cohort

#### Case dataset vs. control dataset

AR shows a statistically significant difference between the subgroups when considered as continuous variable (mean case dataset: 13.21%; mean control dataset: 43.21%, *p* <  0.001, see Table 3).

Moreover, considered as dichotomized variable, different expression of AR among the two populations emerges (*p* = 0.003 for10% cut-off and *p* <  0.001 for IRS). Thus, odds-ratio (OR) was evaluated for 10% AR’s cut-off and the risk to develop a brain metastasis was 8,33 times higher in women with AR-negative primary EOCs (CI 95%: 2.15–32.29). (See Table 4).

### Combined analysis

We analyzed the combined dataset including previous published cohort and new validation cohort, for a total of 40 cases and 40 controls.

#### Case dataset

Median age at diagnosis was 57 years (range 39–76); 34/40 cases were categorized as serous carcinomas (85%) and all cases were considered as high grade (G3) tumors (40/40, 100%). As for FIGO classification, 4 cases (10%) were stage II, 25 (62.5%) stage III and 11 (27.5%) stage IV.

Most of our cases (31/40, 77.5%) were treated with up-front surgery; nine (22.5%) received neo-adjuvant chemotherapy (NACT) followed by Interval Debulking Surgery (IDS). Each case received a platinum-based chemotherapy, with Carboplatin alone (2/40, 5%) or in combination with paclitaxel, (38/40, 95%). Residual tumor was present in 17 cases (42.5%), absent in 14 (22.5%), unknown for 9 patients (22.5%).

All cases relapsed, with a median PFS of 18 months (range 0–62). Median age at diagnosis of CNS involvement was 59.5 years, with a median bPFS of 25 months (range 0–87 months). Twenty one out of 40 (52.5%) patients developed brain metastases as the first site of relapse. Further sites of first relapse were lymph nodes and/or peritoneum (14/40, 35%) lung or liver (5/40, 12.5%).

Overall, median bOS was 12 months (range 2–112), while median OS was 47.5 months (range 4–173). When statistical analyses were performed (May 2018), 10/40 patients (25%) were alive, whilst 29 (72.5%) had died; vital status data was not available for one patient (2.5%).

Table 5 shows the most relevant clinico-pathological parameters of case subgroup.

**Table 5:** Clinico-pathological features of primary ovarian lesion of combined dataset: case dataset vs control dataset

**Clinico-histopathological features**	**Case Dataset** ***n*** **= 40 (%)**	**Control Dataset** ***n*** **= 40 (%)**	***p*Value**
**Age, median (years) [range]**	57 [39–76]	63.5 [36–78]	0.960
**Histological type**			
Serous	34 (85)	40 (100)	0.262
Clear cell	2 (5)		
Endometrioid	1 (2.5)		
Mucinous	1 (2.5)		
Squamous	1 (2.5)		
Undifferentiated	1 (2.5)		
**Histological grade**			
G3	40 (100)	40 (100)	1
**FIGO stage**			
II	4 (10)	4 (10)	1
III	25 (62.5)	25 (62.5)	
IV	11 (27.5)	11 (27.5)	
**Type of surgery**			
Upfront	31 (77.5)	18 (45)	0.003
Neoadjuvant CT + IDS	9 (22.5)	22 (55)	
**Macroscopic residual tumor**			
Present	17 (42.5)	19 (47.5)	0.611
Absent	14 (35)	20 (50)	
Not available	9 (22.5)	1 (2.5)	
**First-line chemotherapy**			
Platinum-based	40 (100)	40 (100)	1
**Relapse**			
Present	40 (100)	30 (75)	0.001
Absent		10 (25)	
**First site of relapse**			
CNS	21 (52.5)		< 0.001
Lymph nodes and / or peritoneum	14 (35)	28 (93.3)^a^	
Other	5 (12.5)	2 (6.7)^a^	
**Patient’s status**			
Alive	10 (25)	21 (52.5)	0.015
Dead	29 (72.5)	19 (47.5)	
Not available	1 (2.5)		
**PFS, median (months) [range]**	18 [0–62]	17.5 [5–73]	
**OS, median (months) [range]**	47.5 [4–173]	40 [6–101]	

*IDS* Interval Debulking Surgery, *CT* Chemotherapy, *CNS* Central Nervous System, *PFS* Progression Free Survival, *OS* Overall Survival

^a^Disease progression did not occur for 10 patients

For more details about features of CNS involving see Table 2.

#### Control dataset

This group included 40 women with a median age of primary EOC diagnosis of 63.5 years (range 36–78). All of our controls were identified as high grade (G3) serous carcinomas (40/40, 100%). There were 4 FIGO stage II (10%), 25 FIGO stage III (62.5%) and 11 FIGO stage IV (27.5%). Most patients (22/40, 55%) were treated with neoadjuvant chemotherapy (Carboplatin alone, 1/22 4.5%, Carboplatin and paclitaxel, 19/22 86.4%, Carboplatin, paclitaxel and Bevacizumab, 2/22 9.1%) followed by IDS. Up-front surgery was chosen for the remaining 18 women, followed by adjuvant chemotherapy (Carboplatin plus paclitaxel, 13/18 72.2% Carboplatin, paclitaxel and Bevacizumab, 3/18 16.7%, Carboplatin and Pegylated Liposomal Doxorubicin 1/18 5.6%, Carboplatin, Pegylated Liposomal Doxorubicin and Bevacizumab, 1/18 5.6%). Nineteen patients (47.5%) had residual tumor after surgery; for one patient (1/40, 2.5%) this datum was not retrievable. Thirty out of 40 controls (75%) experienced at least one relapse of the disease, whose first localization involved either lymph nodes and/or peritoneum (28/30, 93.3%) or liver (2/30, 6.7%). Median PFS was 17.5 months (range 5–73), whereas median OS was 40 months (range 6–101). When data were processed, 19 (47.5%) patients had died.

Table 5 shows the most relevant clinico-pathological parameters of control subgroup of combined dataset.

Cases and Controls proved to be comparable populations: no statistical difference was observed for age (*p* = 0.960), histotype (*p* = 0.262), tumor grade (*p* = 1), FIGO stage (*p* = 1), residual tumor after surgery (*p* = 0.611) and first line chemotherapy (*p* = 1) (Table 5). There were significant differences between cases and controls, in particular upfront surgery vs interval cytoreductive surgery, incidence of relapse and number of surviving patients.

For AR protein expression of combined cohort as continuous variable of primary vs metastatic lesions and of cases vs controls see Table 6. AR expression in cases and controls dataset of combined cohort considered as dichotomized variable is showed in Table 7.

**Table 6:** Immune-histochemical results and statistical analyses of AR considered as continuous variable: comparisons of both cases vs controls and primary vs metastatic lesions

**IHC parameter**	**Cases vs controls // primary vs metastatic lesions (%)**	**N**	**Mean (%)**	**Median (%)**	**Range**	***p*Value**
**AR**	Cases	39	14.15	5	0–70	**< 0.001**
	Controls	40	42.05	37.5	0–95	
	Primary tumor	24	12.70	2.5	0–70	0.270
	Brain metastasis	24	9.25	0	0–60	

*AR* Androgen Receptor

**Table 7:** Immune-histochemical results and statistical analyses of AR considered as dichotomized variable as for both cases and controls datasets

**IHC parameter**	**Cut-off**	**Case dataset (primary ovarian lesions) (%)**	**Control dataset (primary ovarian lesions) (%)**	***p*Value (Cases vs controls: IHC comparison)**	**OR (Cases vs controls) (CI 95%)**	
**AR**	< 1%	7/39 (17.9)	2/40 (5)	0.087		
	≥ 1%	32/39 (82.1)	38/40 (95)			
	< 10%	26/39 (66.7)	7/40 (17.5)	**< 0.001**	**9.429 (3.290–27.020)**	
	≥ 10%	13/39 (33.3)	33/40 (82.5)			
	IRS ≤ 2	31/39 (79.5)	13/40 (32.5)	**< 0.001**		
	IRS > 2	8/39 (20.5)	27/40 (67.5)			

*AR* Androgen Receptor

### AR expression is significantly reduced in cases vs controls’ combined cohort

#### Case dataset vs. control dataset

AR shows a statistically significant difference between cases and controls when considered as continuous variable (mean case dataset: 14.15%; mean control dataset: 42.05%, *p* < 0.001, Table 6).

Considered as dichotomized variable, the two populations showed different expression of AR (*p* < 0.001 for both 10% cut-off and IRS). Odds-ratio (OR) evaluated for 10% AR’s cut-off was 9.43 (CI 95%: 3.290–27.020) (See Table 7).

**Fig. 1:**
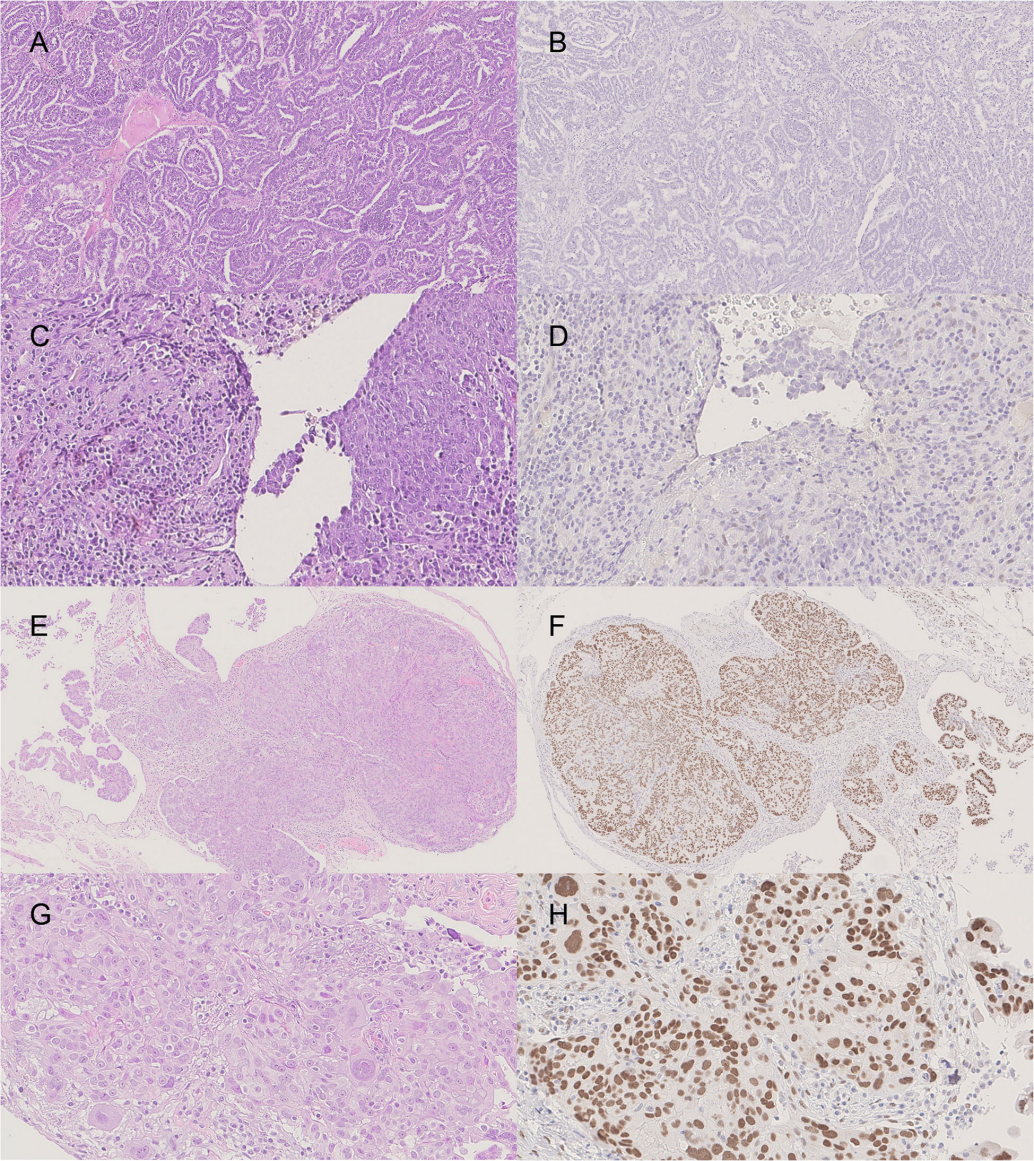
**a** (5x) **c** (20x): H&E staining in ovarian serous carcinoma metastatic to the brain with the corresponding negative AR immunoreaction (**b**, 5x) and focal and weak AR expression (**d**, 20x) in primary tumor cells. **e** (5x), **g** (20x): H&E staining in control cases of ovarian carcinoma with strong and diffuse AR expression (**f, h**: 5x and 20x respectively)

Figure 1 shows the immune-histochemical expression of AR with the corresponding H&E staining in two representative cases and two controls.

### AR expression is not differentially expressed in primary ovarian cancers vs paired brain metastases

#### Primary ovarian cancers vs paired brain metastases

IHC expression of AR doesn’t exhibit statically significant difference of expression between the two groups in the combined population, considering continuous variable only (See Table 6).

### Kaplan-Meier’s curves of combined population

**Fig. 2:**
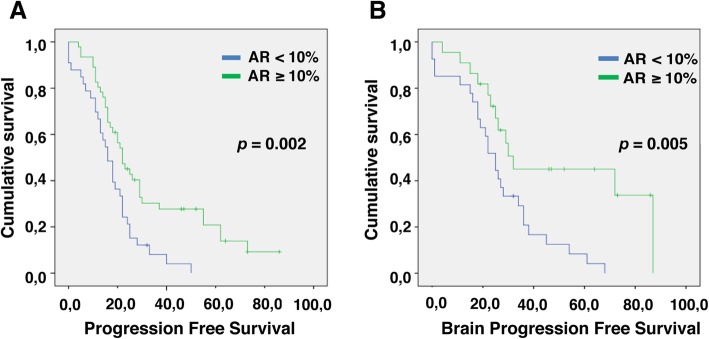
Progression Free Survival in relation to AR 10% (curve **a**, *p* = 0.002); Brain Progression Free Survival in relation to AR 10% (curve **b**, *p* = 0.005)

Survival curves were obtained using PFS (progression free survival) and bPFS (progression brain metastasis free survival) as events of interest in the combined population. AR results associated with prognosis in this population as show the Kaplan-Meier’s (KM) curves, in particular: KM curve by AR 10% and PFS (Fig. 2, curve a, *p* = 0.002) and KM curve by AR 10% and bPFS (Fig. 2, curve b, *p* = 0.005).

## Discussion

Little evidence from small series of patients exists about predictors of CNS involvement from EOC. Few clinical and molecular factors have been studied, such as CD133 overexpression and platinum resistance [17], overexpression of MDR-1 (multi drug resistance 1) [18], having suffered from a previous breast cancer [19], loss of BRCA function [20] and, presence of mutations in BRCA1 and BRCA2 genes [21]. In our work we explored the predictive and prognostic role of AR.

The main conclusion of the current study is that decreased AR levels predict CNS involvement from EOC. In an independent validation cohort, we showed a 8,33 times higher risk of disease’s spreading to CNS if AR’s amount on primary tumor is lower than 10%, statistically significant both for AR considered as dichotomized or continuous variable (see Table 4). Moreover, in our complete dataset, including cases and controls of previous work [14], we confirmed these data, with AR expression significant reduction in cases vs controls (see Tables 6 and 7) and a more than 9 times greater risk of developing brain metastases if AR showed less than 10% of expression (see Table 7).

In combined dataset AR levels also have a prognostic value, as reported in KM curve (Fig. 2, curve a). Indeed, a reduced AR, is associated with shorter PFS, regardless of site relapse.

Our data are consistent with other studies performed in EOC [16, 22, 23] breast [24, 25] and endometrial cancer [26] that suggest a positive prognostic role of AR expression.

Most importantly we show for the first time that besides causing earlier involvement of any other site, AR loss also has a negative prognostic relevance if bPFS is considered: lower AR levels correlate with an earlier CNS spreading (Fig. 2, Curve b).

Moreover, we also first compare AR expression in primary and paired CNS metastatic site (See Table 6).

Data on this topic are limited [27, 28]. When this receptor is considered as continuous variable, no difference of expression is observed between ovarian and brain lesions, differently from the previous study [14] (see Table 6); it is clear that a correlation to de-differentiation exists, since metastatic lesions disclose lower percentages of the evaluated protein compared with primary ovarian tumors.

It is worthwhile mentioning that 21 cases (52.5%) of the combined population had the CNS as the first site of relapse. Such tendency may be explained with the improvement of chemotherapeutic agents, able to contrast typical routes of metastatic spread, though unable to cross the blood-brain barrier (BBB) [29].

Our study has some limitations. First, sample size was increased, but is still relatively small. Second, BRCA mutational status was known only in 4/40 (10%) of cases (3 were mutated, one was not). This lack of information may be important since BRCA mutation is a known independent positive predictive and prognostic factor [30]. Third, in 22.5% of patients residual disease after surgery is unknown. This is a limit because macroscopic residual tumor after surgery is an independent prognostic factor in ovarian cancer [31], although it is unlikely to impact specifically on the development of brain metastases. Fourth, nearly all patients in our study have serous cancers, a good prognostic factor for patients with CNS involvement [11]. We could have selected a population with better prognosis, but in any case it appears to be well balanced to the control group as regards histology (see Table 5).

In conclusion, we confirmed in an independent validation cohort that AR reduction is predictive of CNS involvement from EOC. Assessment of AR levels might help in the identification of high risk patients who may benefit from dedicated follow up procedures to anticipate diagnosis and treatment.

However, before applying this biomarker to clinical practice, further evidence on larger series of EOC patients is needed.

## Data Availability

The datasets used and analysed during the current study are available from the corresponding author on reasonable request.

## Abbreviations

CNS: Central nervous system
EOC: Epithelial Ovarian Cancer
AR: Androgen Receptor
IHC: Immunohistochemistry
PFS: Progression free survival
bPFS: Progression brain metastases free survival
OS: Overall survival
bOS: Brain metastases overall survival
H&E: Haematoxylin and Eosin
IRS: Immunoreactive score
IDS: Interval Debulking Surgery
NACT: Neo-adjuvant chemotherapy
CT: Chemotherapy
WBRT: Whole Brain RadioTherapy
SRS: Stereotactic RadioSurgery
pCR: Pathological complete response
KM: Kaplan-Meier
MDR-1: Multi drug resistance 1
BBB: Blood-brain barrier
